# Identification of new HIV-1 Gag-specific cytotoxic T lymphocyte responses in BALB/c mice

**DOI:** 10.1186/1743-422X-5-81

**Published:** 2008-07-14

**Authors:** Silvia Cellini, Cinzia Fortini, Eleonora Gallerani, Federica Destro, Egidio Brocca Cofano, Antonella Caputo, Riccardo Gavioli

**Affiliations:** 1 Department of Biochemistry and Molecular Biology, Via L. Borsari 46, University of Ferrara, 44100, Ferrara, Italy; 2 Department of Histology, Microbiology and Medical Biotechnologies, Via A. Gabelli 63, University of Padova, 35121, Padova, Italy

## Abstract

**Background:**

As HIV-specific cytotoxic T cells play a key role during acute and chronic HIV-1 infection in humans, the ability of potential anti-HIV vaccines to elicit strong, broad T cell responses is likely to be crucial. The HIV-1 Gag antigen is widely considered a relevant antigen for the development of an anti-HIV vaccine since it is one of the most conserved viral proteins and is also known to induce T cell responses. In the majority of studies reporting Gag-specific cellular immune responses induced by Gag-based vaccines, only a small number of Gag T cell epitopes were tested in preclinical mouse models, thus giving an incomplete picture of the numerous possible cellular immune responses against this antigen. As is, this partial knowledge of epitope-specific T cell responses directed to Gag will unavoidably result in a limited preclinical evaluation of Gag-based vaccines.

**Results:**

In this study we identified new Gag CD8+ T cell epitopes in BALB/c mice vaccinated with the HIV-1 Gag antigen alone or in combination with the HIV-1 Tat protein, which was recently shown to broaden T cell responses directed to Gag. Specifically, we found that CTL responses to Gag may be directed to nine different CTL epitopes, and four of these were mapped as minimal CTL epitopes.

**Conclusion:**

These newly identified CTL epitopes should be considered in the preclinical evaluation of T cell responses induced by Gag-based vaccines in mice.

## Background

Cellular immune responses are a critical part of the host defence against viruses, with cytotoxic T lymphocytes (CTLs) playing a key role in recognizing and eliminating infected cells. CTLs identify their targets as 8–10 amino acid long peptides which are derived from the intracellular degradation of viral antigens and presented in association with major histocompatibility complex class I (MHC-I) molecules at the surface of infected cells [1-3].

Several studies have indicated that HIV-specific T cell responses play a key role in limiting the progression of acute and chronic infection in humans [4,5], and that long-term non-progressors have consistently higher levels of HIV-specific T cell responses than progressors [6]. Thus, the ability of potential vaccines for HIV to elicit strong, broad T cell responses is likely to be a determining factor in their success.

We have recently reported that vaccines based on a combination of the HIV-1 Tat protein with heterologous antigens induce broader T cell responses against the co-administered antigen, thereby indicating Tat as a useful tool in the development of novel vaccination strategies against AIDS [7-9].

As the HIV-1 Gag antigen is one of the most conserved viral proteins, and is known to induce T cell responses, both in animal models and in humans, it is widely considered a relevant antigen for the development of an anti-HIV vaccine. Indeed, previous studies have shown that Gag-specific T cell responses contribute to clear primary viremia and control later viral replication, thereby slowing progression of the disease [4,10-12].

Small animal models, in particular mice, represent a useful tool for studying the dynamics of immune responses induced after vaccination, although, the evaluation of cellular responses induced by vaccination is usually restricted to immunodominant T cell epitopes, which represent, only a minor part of the overall cellular immune response. In order to expand our limited knowledge of epitope-specific T cell responses directed to a given antigen, the aim of this study was to identify the repertoire of CD8+ T cell epitopes of the HIV-1 Gag antigen in BALB/c mice vaccinated with the HIV-1 Gag protein.

## Results and Discussion

### *In vivo *modulation of epitope-specific T cell responses against the HIV-1 Gag antigen by the HIV-1 Tat protein

We recently demonstrated in BALB/c mice vaccinated with the HIV-1 Gag protein [9] that Gag-specific T cell responses are directed to 7 different peptides (peptides: 42, 49, 50, 53, 65, 75 and 76). Only two (49 and 50) of these peptides were already known to contain the major K^d^-restricted CTL epitope (AMQ, aa 197–205), while peptides 65 (aa 257–271) and 75 (aa 297–311) were known to contain T cell epitopes which had not been fully characterized [13,14].

**Figure 1:**
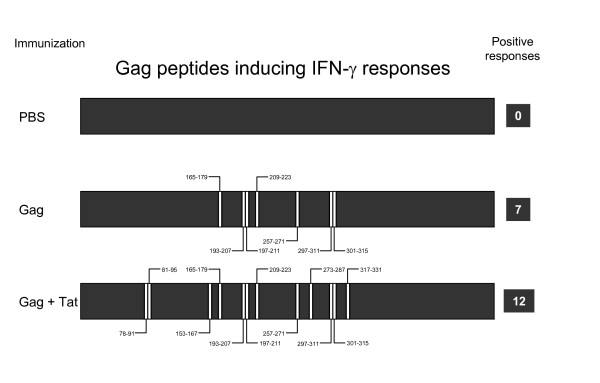
Tat broadens T cell responses against HIV-1 Gag. Mice were immunized with Gag alone, with Gag in combination with Tat protein, or with PBS alone. After 3 immunizations, fresh splenocytes were tested by IFNγ Elispot assay using 15 amino acid long peptides encompassing the entire Gag sequence. Positive responses to the indicated peptides are represented in the Figure.

In a previous study, we also showed that co-immunization of mice with the HIV-1 Tat protein broadens the cellular responses against Gag, as fresh splenocytes purified from mice immunized with Gag and Tat responded to 12 different peptides (20, 21, 39, 42, 49, 50, 53, 65, 69, 75, 76 and 80), five more (20, 21, 39, 69 and 80) than those reported in mice immunized with Gag alone, thereby suggesting that Tat expands T cell responses directed to the Gag antigen [9]. A summary of the previously detected T cell responses directed to 15 amino acid long peptides is reported in Figure 1.

### Identification of new CD8+ T cell responses against the HIV-1 Gag antigen

**Figure 2:**
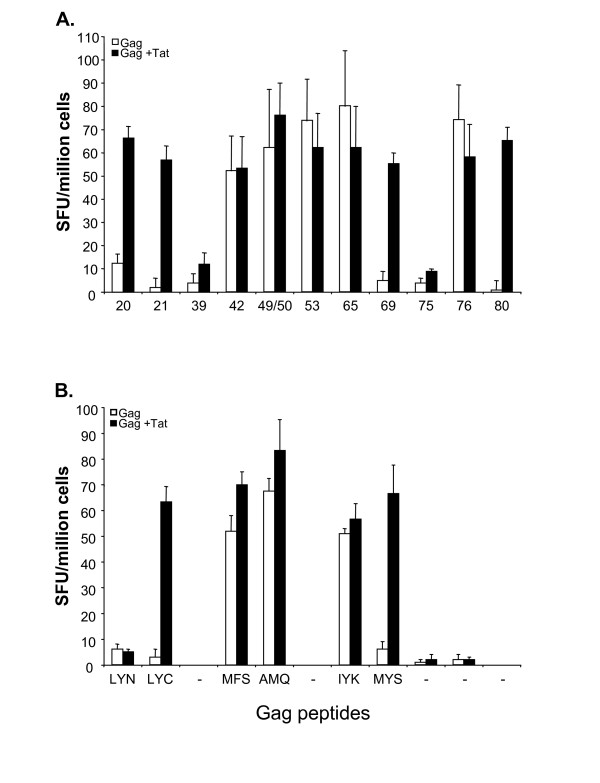
Peptide-specific CD8+ T cell responses against the HIV-1 Gag antigen in mice immunized with Gag or with the combination Gag and Tat. After 3 immunizations, fresh splenocytes were pooled and purified, then CD8+ T cells were tested by IFNγ Elispot assay after stimulation with the indicated 15 mers (panel A), or with the indicated 8–10 mers derived from the above 15 mers (panel B). Results from mice immunized with Gag (empty bars) and from mice immunized with Gag and Tat (filled bars) are expressed as SFU ± SD/10^6 ^cells. The mean +/- SD of three independent experiments is shown.

To characterize peptide-specific CD8-mediated T cell responses in mice vaccinated with Gag alone or with a combination of Gag and Tat, CD8+ T cells were purified from fresh splenocytes and tested by IFN-γ Elispot assays using the previously identified peptides (Figure 1). As shown in Figure 2A, the majority of peptides were found to be targets of CD8+ T cells. Only peptides 39 and 75 failed to activate CD8+ cells, thereby confirming previous results suggesting that peptides 39 and 75 contain two CD4+ T cell epitopes [14].

To identify the minimal CD8 target epitopes, we then used the peptide binding motif for K^d ^class I molecules [15]. By this approach, 6 different potential epitopes within the sequences of the responding 15 mers were identified (Table 1). Eight to ten amino acid long peptides were used to stimulate purified CD8+ cells and to perform IFN-γ Elispot assays. As shown in Figure 2B, CD8+ T cell responses were clearly directed to peptides LYC, MFS, IYK and MYS, contained in 15 mers 21, 42, 65 and 69, respectively. CD8-mediated T cell responses were also directed to the previously described AMQ peptide [13], contained within peptides 49 and 50. Remarkably, the results indicate that peptide 65 contains not only a CD4+ T cell epitope, as previously suggested [14], but also a CD8+ T cell epitope, since purified CD8+ T cells clearly responded to the IYK epitope contained within peptide 65.

**Table 1:** Gag peptides and predicted CTL epitopes

**Peptide ** **number**	**Peptide Sequence**	**aa**	**Predicted ** **CTL Epitope**	**aa**	**Code**
**20**	SLYNTVATLYCVHQR	78–91	LYNTVATL	78–85	LYN
**21**	TVATLYCVHQRIEVK	81–95	LYCVHQRI	85–92	LYC
**39**	NAWVKVVEEKAFSPE	153–167	CD4 epitope	-	-
**42**	SPEVIPMFSALSEGA	165–179	MFSALSEGA	171–179	MFS
**49**	GHQAAMQMLKETINE	193–207	AMQMLKET	197–205	AMQ
**50**	AMQMLKETINEEAAE	197–211	AMQMLKET	197–205	AMQ
**53**	AAEWDRLHPVHAGPI	209–223	Not identified	-	-
**65**	PVGEIYKRWIILGLN	257–271	IYKRWIILGL	261–270	IYK
**69**	IVRMYSPTSILDIRQ	273–287	MYSPTSILDI	276–285	MYS
**75**	VDRFYKTLRAEQASQ	297–311	CD4 epitope	-	-
**76**	YKTLRAEQASQEVKN	301–315	Not identified	-	-
**80**	MTETLLVQNANPDCK	317–331	Not identified	-	-

**Figure 3:**
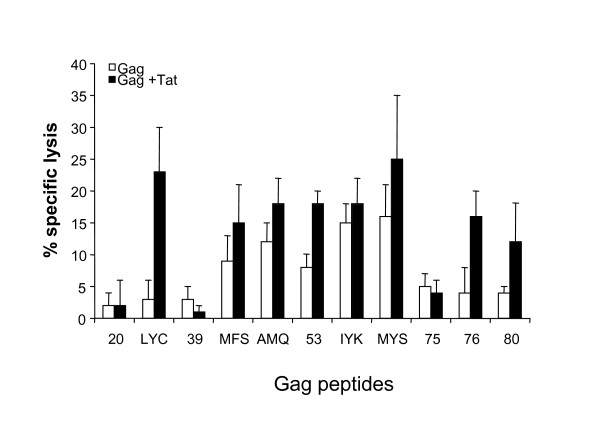
Peptide-specific cytotoxic T cell responses against the HIV-1 Gag antigen in mice immunized with Gag or with the combination Gag and Tat. Splenocytes from each group were re-stimulated *in vitro *with the indicated peptides. After 5 days, T cell cultures were tested against P815 cells (ratio 50:1) treated with the indicated peptides. Results from mice immunized with Gag (empty bars) and from mice immunized with Gag and Tat (filled bars) are expressed as % specific lysis calculated by subtracting the lysis of untreated P815 cells (always below 3%). The mean +/- SD of the results from three independent experiments, performed in triplicate, is shown.

Further characterization of the T cell responses directed to the identified peptides was then carried out by ^51^Cr-release cytotoxicity assays. To this end, splenocytes from mice immunized with Gag and Tat or with Gag alone were stimulated *in vitro *with the identified 15 amino acid long peptides and assayed in cytotoxicity against P815 cells pulsed with the corresponding 15 amino acid long peptides or with the minimal CTL epitopes. As shown in Figure 3, peptide-stimulated T cell cultures specifically lysed cells pulsed with LYC, MFS, AMQ, 53, IYK, MYS, 76 and 80. These results demonstrate that all peptide-specific CD8+ T cells, except CD8+ T cells specific for peptide 20, in addition to secrete IFN-γ are able to lyse peptide-pulsed target cells.

## Conclusion

In this study we have defined new CD8-mediated Gag-specific T cell responses in BALB/c mice vaccinated with the Gag protein alone or with the combination Tat/Gag, which had previously been shown to be very efficient at inducing Th1 responses directed to Gag [9]. We demonstrated that 9 out of 11 peptides stimulating IFN-γ T cell responses were recognized by CD8+ T cells. In addition, CD8+ T cells efficiently lysed peptide-pulsed target cells. Four peptides were also characterized as minimal CTL epitopes.

Induction of HIV-1-specific CTL responses has become a critical component in the design of recombinant subunit vaccines aimed at controlling HIV infection, and these CD8-restricted epitopes, which, with the exception of the AMQ peptide, were all identified here for the first time, should be exploited in preclinical models for a complete evaluation of cellular responses induced by Gag-based vaccines.

## Methods

### HIV-1 proteins

The biologically active HIV-1 Tat protein from the human T lymphotropic virus type IIIB isolate (BH10 clone) was expressed in *E. Coli *and purified by heparin-affinity chromatography and HPLC as previously described [16]. The Tat protein was stored in lyophilized form at -80°C, reconstituted in degassed buffer before use, and handled as described to prevent oxidation and loss of biological activity [17]. Different lots of Tat were used with reproducible results, and in all cases endotoxin concentration was undetectable (detection threshold: 0.05 EU/μg). HIV-1 GagSF2 protein was obtained from the NIH AIDS Reagent Program.

### Peptides

Peptides were synthesized by the solid phase method and purified by HPLC to >98% purity, as previously described [18]. Structural verification was performed by elemental and amino acid analysis and mass spectrometry. Gag peptides, 15 amino acid long and overlapping by 10 to 11 amino acids, spanning the entire Gag (HIV-1 consensus subtype B Gag complete set # 8117) sequence, were provided by the NIH AIDS Reagent Program. Peptides were dissolved in DMSO at 10^-3 ^M, kept at -20°C, and diluted in PBS before use.

### Immunization of mice

BALB/c mice (H-2^d^) (Harlan, Udine, Italy) were immunized subcutaneously at a single site in the back with 5 μg of HIV-1 Gag protein alone or in combination with 5 μg of native monomeric biologically active Tat protein in Freund's adjuvant [9]. Each group was composed of 5 animals. Immunogens were given subcutaneously in 100 μl injections, on days 1, 14 and 28, and mice were sacrificed 10 days after the final boost (day 38). During the course of the experiments, animals were checked twice a week at the site of injection, as well as for their general conditions (such as liveliness, food intake, vitality, weight, motility, sheen of fur). No signs of local or systemic adverse reactions were observed at any time in mice receiving the immunogens, as compared to control or untreated mice. Animal use was carried out according to European and institutional guidelines.

### Splenocyte purification

Splenocytes were purified from spleens squeezed onto filters (Becton Dickinson). Spleens of each experimental group were pooled. Following red blood cell lysis, cells were resuspended in RPMI 1640 supplemented with 10% FBS (Gibco) and immediately used for the analysis of antigen-specific cellular immune responses by cytotoxicity or Elispot assays [9]. When indicated, splenocytes were also stimulated *in vitro *with 1 μg/ml of peptide and tested in cytotoxic assays after 5 days of culture. Purified CD8+ T cells were obtained using the BD™ IMag Mouse Lymphocyte Enrichment Set-DM filters (Becton Dickinson), according to the manufacturer's instructions. Purified cells were stained with FITC-conjugated anti-mouse CD8 mAb (Becton Dickinson). Flow cytometry analysis was performed with a FACScan (Becton Dickinson), and CD8-positive cells were > 90% in all cases.

### Cytotoxicity assay

P815 target cells were labeled with Na_2_^51^CrO_4 _for 90 min at 37°C. Cytotoxicity tests were routinely run in triplicate at different effector:target ratios [18]. Percent specific lysis was calculated as 100× (cpm sample - cpm medium)/(cpm Triton X-100 - cpm medium). Spontaneous release was always less than 20%.

### Elispot assay

Elispot (IFN-γ) was carried out using commercially available kits provided by Becton Dickinson, according to the manufacturer's instructions. Briefly, 96-well nitrocellulose plates were coated with 5 μg/ml of anti-IFN-γ overnight at 4°C [9]. The following day, the plates were washed 4 times with PBS and blocked with RPMI 1640 supplemented with 10% FBS for 2 hours at 37°C. Splenocytes (2.5 × 10^5^/200 μl) were added to the wells (duplicate wells) and incubated with peptides (10^-6 ^M) for 16 hours at 37°C. Controls were represented by cells incubated with Concanavaline A (Sigma; 5 μg/ml) (positive control) or with medium alone (negative control). The spots were read using an Elispot reader (Aelvis, Germany). Responses at least 3 times higher than the mean number of spots in the control wells and ≥ 50 spots/well/10^6 ^cells were defined as positive. Results are expressed as net number of spot forming units (SFU)/10^6 ^cells: [mean number SFU of peptide treated wells minus mean number SFU of the negative control].

## Competing interests

The authors declare that they have no competing interests.

## Authors' contributions

SV, CF and EG performed the immunological assays. FD and EBC performed mice vaccinations and sacrifice. AC and RG conceived the strategies and designed the experiments. SV and RG contributed to data analysis. RG wrote the manuscript. All authors read and approved the final manuscript.
